# Effect of glycemic control on tuberculosis treatment outcomes among patients with tuberculosis and diabetes mellitus: A systematic review and meta‐analysis

**DOI:** 10.1111/tmi.14140

**Published:** 2025-06-23

**Authors:** Maham Zahid, Saima Afaq, Kashif Shafique, Fatima Khalid Qazi, Urooj Ashfaq, Muhammad Asim, Shaista Nooreen, Sofia Shehzad

**Affiliations:** ^1^ Institute of Public Health & Social Sciences Khyber Medical University Peshawar Pakistan; ^2^ Department of Health Sciences University of York York UK; ^3^ School of Public Health Dow University of Health Sciences Karachi Pakistan; ^4^ Sardar Begum Dental College Gandhara University Peshawar Pakistan

**Keywords:** blood glucose, diabetes, glycaemic control, treatment failure, tuberculosis

## Abstract

**Introduction:**

Tuberculosis (TB) and diabetes mellitus comorbidity can lead to poor TB treatment outcomes, particularly with uncontrolled blood glucose levels. Understanding the impact of glycemic control on TB treatment outcomes is essential.

**Objective:**

To synthesise evidence on the association between glycemic control and TB treatment outcomes in patients with TB and diabetes mellitus.

**Methodology:**

A systematic review was conducted using Medline, Embase, Scopus, Web of Science, Cumulative Index to Nursing and Allied Health Literature (CINAHL) and Google Scholar for all types of studies published between 1975 and May 2024, including adult TB patients of >18 years of age, with or without diabetes mellitus for whom blood glucose testing along with TB treatment outcome comparison with glucose levels (low/high) was reported were considered for inclusion. A random‐effects model was used for meta‐analysis, heterogeneity was assessed using *I*‐squared statistics, subgroup and sensitivity analysis was performed followed by publication bias assessment.

**Results:**

Of 576 identified studies, 12 met the inclusion criteria, analysing 2320 cases (1572 with uncontrolled high blood glucose [≥7% HbA1c] and 748 with controlled low blood glucose [<7% HbA1c]). Low certainty evidence shows that patients with uncontrolled high glucose had a 1.91 times higher risk of TB treatment failure (risk ratios [RR] = 1.91, 95% confidence interval [CI] 1.81–3.07, *p* = 0.008), and a 2.97 times higher risk of sputum positivity at 3 months (RR = 2.97, 95% CI 1.10–8.07, *p* = 0.03). Subgroup and sensitivity analyses showed significant improvement in pooled effects, lowering of heterogeneity and narrower CIs. For overall pooled effect, substantial heterogeneity was observed; therefore, the interpretation and generalisation of results should be done with caution.

**Conclusion:**

A low certainty evidence shows that uncontrolled high blood glycemic level significantly impacts TB treatment outcomes, increasing treatment failure and sputum positivity among TB patients with diabetes mellitus.

## INTRODUCTION

Tuberculosis (TB) remains a significant global health challenge, affecting millions and imposing a substantial burden on public health systems worldwide, and especially in developing countries [1]. It is estimated that one‐quarter of the global population harbours latent TB infection, with 10.6 million new cases and 1.6 million deaths reported in 2022 [2]. For the 8.5 million individuals surviving incident TB in 2019, an estimated 58 million post‐TB disability‐adjusted life years (DALYs) will accrue over their lifetime, nearly half of the 122 million DALYs attributed to TB overall [3]. Numerous risk factors have been identified that exacerbate TB progression and outcomes, including diabetes mellitus (DM) [4].

DM is a critical risk factor for TB known to exacerbate the disease and treatment prognosis [5]. Individuals with DM are reported to have a two‐ to four‐fold higher risk of developing active TB compared to non‐diabetic individuals [6, 7]. As per the World Health Organisation (WHO) report, around 0.4 million people suffered from TB‐diabetes multimorbidity in the year 2021 [8]. DM not only elevates the risk of TB incidence but is also associated with unfavourable TB treatment outcomes [9], including delayed sputum culture conversion, treatment failure, disease relapse (odds ratios [OR] 1.64), the emergence of drug resistance (OR 1.98) and increased mortality (OR 1.88) [10]. Hyperglycaemia or failure to maintain normal glucose levels during TB treatment period is thought to compromise immune responses, impairing the host's ability to combat TB infection effectively [11, 12, 13, 14].

The International Union against Tuberculosis and Lung Disease has highlighted the potential benefits of glycemic control in improving TB treatment outcomes among patients with DM. However, evidence on this relationship remains limited and inconsistent [15]. Two systematic reviews have examined this phenomenon [16, 17], but both have limitations. One of the reviews was published in 2017 (more than 5 years ago), while the other was published recently in 2024 [17], but included studies only up to 2017, limiting incorporation of more recent evidence on the importance of glucose control in TB patients. There are at least five relevant studies published between 2020 and 2024 that were identified in the current updated review. Additionally, although the review used weighted random‐effects models, it did not report any subgroup or sensitivity analyses, reducing the robustness of its findings. Heterogeneity was generically interpreted and the publication bias was not reported properly. This warrants the need to update the existing systematic reviews to fill in the existing gap to reflect complete and accurate evidence contributing towards the importance of glucose control in diabetic TB patients. This systematic review aims to address these gaps by incorporating the most recent and robust evidence to assess the impact of glycemic control on TB treatment outcomes in patients with DM. By employing advanced statistical methods and synthesising updated research, this review seeks to provide actionable insights that can inform targeted public health strategies and improve clinical outcomes for patients with coexisting TB and DM.

## METHODS

The protocol of this review was developed in compliance with Preferred Reporting Items for Systematic Reviews and Meta‐Analyses Protocols (PRISMA‐Pidelines and was registered with the International Prospective Register of Systematic Reviews (PROSPERO) registry (CRD42024539451). Findings are reported following the Meta‐Analyses of Observational Studies in Epidemiology (MOOSE) and Preferred Reporting Items for Systematic Reviews and Meta‐Analyses (PRISMA) guidelines (Appendixes 1 and 2, Supporting Information S1) [18].

### Eligibility criteria for inclusion

Experimental (randomised controlled trials [RCTs], quasi‐experimental designs) or observational (cross sectional, case–control, cohort) studies published between 1975 and May 2024, including adult TB patients of >18 years of age, with or without DM for whom blood glucose testing (at any time, including at the start of TB treatment, during treatment or end of treatment at 6 months) along with TB treatment outcome comparison with glucose levels (low/high) was reported, were considered for inclusion. While both pulmonary and extrapulmonary TB studies were considered, those focusing exclusively on extrapulmonary TB or lacking comparisons of TB treatment outcomes as per blood glucose levels were excluded.

### Data sources and search strategy

A systematic search was conducted across multiple databases, including Medline, Embase, Scopus, CINAHL, Web of Science and Cochrane Central, using a combination of Medical Subject Headings (MeSH), index terms, free‐text keywords and search strings from prior systematic reviews. A health information specialist validated and optimised the search strategy, developed in alignment with the Population, Exposure, Comparator, Outcomes, Study Design (PECOS) framework [19]. The finalised search strings are detailed in Table S1. Grey literature was identified via Google Scholar, complemented by hand‐searching and citation mining to ensure comprehensive coverage.

### Selection process

Search results were imported into the Cochrane Covidence platform for screening and management. After automated removal of duplicates, two reviewers independently screened titles and abstracts, with discrepancies resolved by a third reviewer. Full‐text articles of shortlisted abstracts were retrieved and independently assessed for eligibility by two reviewers, with conflicts resolved by a third reviewer. Authors of restricted‐access studies were contacted to obtain full‐text versions.

### Data extraction and quality assessment

Data extraction and quality assessment were conducted independently by two reviewers, with conflicts resolved by a third to ensure accuracy. A standardised tool, adapted from the Covidence Data Extraction Template II, captured predefined data items, including author details, study objectives, population, design, sample size, demographic characteristics, exposure measurements (blood glucose tests performed at baseline, during treatment or end of treatment), TB outcomes (cure rate, treatment failure rate), reported deaths, sputum conversion rates and development of multidrug‐resistant (MDR). Outcomes in the included studies were primarily reported as the number and proportion of events—such as cure, treatment failure, death, sputum conversion and MDR TB—among TB‐DM patients stratified by HbA1c levels (<7% indicating good control, ≥7% indicating poor control). For each outcome, we extracted the number of events along with the corresponding denominators. Authors of studies with incomplete data were contacted, and studies lacking essential information were excluded.

The quality of included observational cohort studies was assessed using the Newcastle‐Ottawa Scale (NOS) [20], evaluating methodological rigour across three domains: selection, comparability and outcomes. The selection domain assessed cohort representativeness, exposure ascertainment and baseline outcome absence. Comparability focused on controlling confounders, while the outcome domain addressed outcome assessment, follow‐up duration and completeness. Studies were classified as good, fair or poor based on the total score derived from these domains.

### Outcomes and exposure measurements

The TB treatment outcomes are classified by the WHO into five categories: cure, treatment complete, failure, loss to follow‐up, not evaluated and death [21] (Table S2). In this review, the primary outcomes included ‘Cure’ and ‘Failure’ while secondary outcomes included ‘sputum conversion’ and ‘deaths.’ The sputum conversion is defined as conversion of positive sputum culture at baseline to negative at 3 or 6 months of treatment. The included studies reported the same outcomes following the same guidelines.

In this review, the exposure was defined as ‘uncontrolled high HbA1c’ with HbA1c >7% at baseline, during or at the end of TB treatment. The unexposed group ‘controlled low HbA1c’ was defined as HbA1c ≤7% at same timepoints. Although the Union guidelines recommend an HbA1c cut‐off of 8% for TB patients with DM [15], the included studies used a 7% cut‐off, which was adopted for this review.

### Statistical analysis

The meta‐analysis was conducted using RevMan [22] (version 5.4) and R studio software. Initially, the included studies characteristics were summarised and tabulated, followed by data extraction using pre‐specified performa. Extracted data were synthesised to assess the pooled association between blood glucose control and TB treatment outcomes, including treatment failure, cure rates, sputum conversion at 3 months, multidrug resistance and mortality. These outcomes were expressed as ORs and risk ratios (RRs) with 95% confidence intervals (CIs), presented in forest plots.

The meta‐analysis employed the weighted inverse variance method using a random‐effects model. Heterogeneity among studies was assessed using the *I* ^2^ statistic, chi‐square test and associated *p*‐values. Publication bias was evaluated visually by inspecting asymmetric distribution in the funnel plots and statistically by using Egger's test. Due to inconsistent stratification across studies, subgroup analysis based on Population, Intervention, Control and Outcome (PICO) characteristics was not feasible. None of the studies reported outcomes stratified by age or sex. The subgrouping was based on the method used to assess exposure and outcome variables, that is, either relying on medical records or longitudinally performing blood testing, and studies in which adjusted results were reported or not reported. Sensitivity analyses were conducted by excluding low‐quality studies and those identified as major contributors to heterogeneity; however, this approach may improve the precision of pooled estimates, it does not fully eliminate the underlying uncertainty.

## RESULTS

### Search results

**FIGURE 1:**
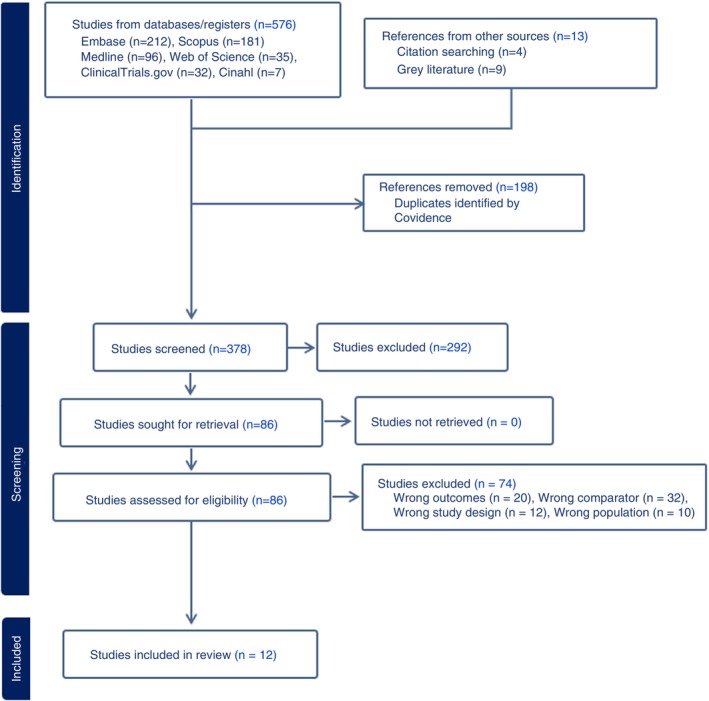
PRISMA flow diagram showing process of study selection.

Initial database search resulted in 576 titles and abstracts that were uploaded in confidence; after duplicate removal, 378 titles and abstracts were screened for inclusion. Finally, there were 12 articles included in the systematic review and meta‐analysis. Although experimental studies were also eligible to be included, none succeeded in fulfilling the inclusion criteria. The study selection process is summarised in Figure 1 as a PRISMA flow diagram, which also outlines the reasons for exclusion at the full‐text review stage.

### Characteristics of included studies

Out of 12, there were 4 (33.3%) studies conducted in India, followed by 2 (16.6%) each in Korea and Peru, and 1 (8.3%) each in China, Iran, Nepal and Taiwan. There were 5 (38.5%) additional studies included in the review that were not included in the latest previous review published in 2024 [17], four of which were published between 2020 and 2024 while the remaining one study was published between 2012 and 2017. Similarly, there were eight (66.6%) cohort studies conducted prospectively assessing the exposure and outcome, while four (33.3%) utilised the previous medical records. All included studies diagnosed DM according to American Diabetes Association (ADA) guidelines, using fasting blood glucose (FBG) and HbA1c levels [23]. Patients were classified as previously diagnosed (via medical records) or newly diagnosed during the studies. Blood glucose levels were measured at least twice: at the start of TB treatment and during or at the end of treatment. Characteristics of individual studies were summarised in detail in Table 1.

**TABLE 1:** Summary of study characteristics for all included studies in the review (*n* = 12).

Author, year	Country, duration	Design	Objective	Population	*n*, age, % males	Exposure measured	Comparison	Funding
Calderon et al. [24], 2022	Peru, 2017–2019	Prospective cohort study	To assess whether persistent deglycation is associated with un favourable TB treatment outcomes	Adult patients >18 years with PTB, with or without DM	Total = 125, TB with DM = 58, median age 47.3 years, 56.8% male	HbA1c (taken at baseline, 2 months, 6 months)	Persistent dysglycemia (HbA1c ≥7% at two time points) versus non persistent dysglycemia (HbA1c <7% at one time)	National Council of Science
Chiang et al. [25], 2015	Taiwan, 2005–10	Retrospective cohort study	To assess the influence of DM, glycemic control and DM‐related comorbidities on manifestations and outcome of treatment of PTB	Confirmed PTB, and patients who (1) were treated with insulin or oral drugs, (2) assign DM‐related International Classification of Disease (ICD) or (3) had a history of DM	Total = 1473, TB with DM = 725, outcomes reported in = 510, <35 years (2.0%) 45–54 years (23.6%), 55–64 years (19.4%), 65–74 years (19.4%), >75 years (20.4%), 77.7% male	HbA1c (value reported in records within 3 months of treatment)	Diabetic TB patients divided into three groups: HbA1c <7%, HbA1c 7–9% and HbA1c >9%	Taiwan Centres of Disease Control
Ghanta et al. [26], 2023	India, 2021–2022	Prospective case–control study	To evaluate the difference in treatment outcome anti‐TB regimens in diabetic patients compared to non‐diabetic patients.	Patients of active TB with known or new DM on screening (as per ADA guideline) as cases and 50 TB patients without DM as control	Total = 100, TB with DM = 50, mean age 48.74 ± 14.33y, gender not reported	HbA1c (taken at end of TB treatment)	Mean HbA1c in good outcome groups versus mean HbA1c in bad outcome group	Self‐funded
Magee et al. [27], 2013	Peru, 2005–2008	Prospective cohort study	(1) Describe the characteristics of TB patients with and without DM; (2) describe DM clinical care among patients with TB and DM; (3) determine DM characteristics associated with favourable TB treatment outcomes.	Patients with suspected or confirmed TB were prospectively enrolled, DM was identified by patient history record.	Total = 1671, TB with DM = 186, outcome reported = 136, 25–44 years (17.7%), 45–64 years (67.2%), >64 years (15.1%), 61.3% male	FBG and HbA1c. Values taken from medical records	Controlled versus not controlled/not mentioned (from medical records)	US Center for Disease Control (CDC), Bill and Melinda Gates and the Infectious Diseases Society of America
Mahato et al. [28], 2023	Nepal	Prospective cohort study	To assess the effect of DM on treatment outcomes of TB patients in Nepal	Newly diagnosed TB patients, with or without DM	Total = 401, TB with DM = 95, Outcome reported = 95, <45 years (64.6%), ≥45 years (35.4%), 62.1% male (age and gender not reported separately for TB‐DM subgroup)	HbA1c (taken at baseline, during and end of treatment)	HbA1c level <7% at 6 months versus HbA1c level ≥7% at 6 months	None declared
Mahishale et al. [29], 2017	India, 2012–2014	Hospital‐based prospective study	To understand impact of poor glycemic control pre‐treatment on the severity of TB and TB treatment outcome in newly diagnosed PTB patients with type‐2 DM	Newly diagnosed smear‐positive PTB patients with type‐2 DM	TB with DM = 630, outcome reported = 630 mean age 48.58 years, 67.1% male	HbA1c, Random Blood Glucose (RBG), FBG (taken at baseline)	Poorly controlled diabetes (HbA1c ≥7%) versus optimal control diabetics (HbA1c <7%).	None declared
Mi et al. [30], 2013	China, 2011–2012	Retrospective cohort study	To describe TB patient in relation to presence or absence of DM and quality of DM control: (i) demographic characteristics (ii) sputum conversion at 2 months (iii) treatment outcomes of new patients with PTB	Patients newly diagnosed with PTB, and screening for history of DM and fasting blood glucose test as mentioned in records	Total = 1589, TB with DM = 189, outcome reported = 86, 15–34 years (8.5%), 35–54 years (47.6%), >55 years (43.9%), 76.7% male	FBG (at baseline, 2 months and 6 months mentioned in records)	DM good control FBG ≤7.0 mmol/L, versus DM poor control FBG 7–10 mmol/L and DM bad control FBG >10 mmol/L.	Department for International Development, UK
Nandhakumar et al. [31], 2013	India, 2010–2011	Retrospective record review	To evaluate the completeness of records of DM and its control in adult TB patients, to determine the association of DM and its control with TB treatment outcome	Treatment cards of all TB cases above the age of 14 years, registered under Revised National TB Control Program (RNTCP)US in Malappuram District of Kerala state, India	Total = 3116, TB with DM = 667, outcome reported = 240, mean age 46.5 ± 17.0 years, 79% male	FBG, RBG (at least one test during treatment assessed as known control status)	Diabetic control as yes (FBG ≤100 mg/dL) versus no (FBG >100 mg/dL)	US Agency for International Development (USAID)
Park et al. [32], 2012	Korea, 2005–2009	Retrospective cohort study	To determine whether control status of DM influences clinical and radiographic manifestations and treatment response in patients with TB	All new culture‐confirmed PTB patients who started anti‐TB medication	Total = 492, Tb with DM = 124, outcome reported = 99, median age 66.0 years, 73.4% male	HbA1c (reported in records prior to diagnosis of TB)	Controlled DM (HbA1C <7.0%) versus uncontrolled DM (HbA1c ≥7.0%)	None mentioned
Tabarsi et al. [33], 2014	Iran, 2012–2013	Prospective cohort study	To examine HbA1c in new TB patients at start and 3 months after TB treatment, and to relate measurements to whether patients successfully completed treatment	All adult patients hospitalised with newly diagnosed PTB, HbA1c assessed at baseline and 3 months later	Total = 317, those with two hbA1c readings = 158, TB with DM = 91, outcome reported = 91, mean age 52.83 ± 21.24 years, 48.73% male	HbA1c (at baseline and 3 months)	Elevated‐normal (HbA1c ≥6.5% at baseline, <6.5% at 3 months) versus normal‐elevated (HbA1c <6.5% at baseline, ≥6.5% at 3 months), elevated‐elevated (HbA1c ≥6.5%)	None mentioned
Udaykumar et al. [34], 2022	India, 2017–2019	Cohort study with a nested case–control study design	(a) to compare TB outcomes with/without DM treatment supervision (b) to compare HbA1c levels to assess control (c) to find association between DM control and TB treatment outcome.	Adult TB‐DM patients routinely diagnosed and consecutively registered in programme were included. Patient on immunosuppressive therapy, Human Immunodeficiency Virus (HIV), pregnant, lactating mothers and lost to follow‐up were excluded	TB with DM = 102, outcome reported = 100, <65 years (76%), >65 years (24%), 80% male	HbA1c and FBG (taken at baseline, 3 month and 6 months)	HbA1c diabetic range (≥7%) versus HbA1c normal range (<7.0%)	State TB Office, Bengaluru
Yoon et al. [35], 2017	Korea, 2012–2014	Multicentre prospective cohort study	To evaluate whether the status of DM control influences the clinical manifestations and treatment responses of PTB	Newly diagnosed PTB patients, divided into three groups: PTB without DM, PTB with controlled DM and PTB with uncontrolled DM	Total = 661, TB with DM = 157, median age 59.0 years, 82.1% male	HbA1c (taken at baseline and 3 months)	Controlled DM (HbA1c <7.0%) versus uncontrolled DM (HbA1c ≥7.0%)	Korea Centres for Disease Control and Prevention and Korean Institute of TB

Abbreviations: DM, diabetes mellitus; FBG, fasting blood glucose; PTB, pulmonary TB; TB, tuberculosis.

The five additional studies [24, 26, 28, 33, 34] in this review included study conducted by Tabarsi et al. [33], (Iran) conducted a hospital‐based study comparing TB outcomes across four HbA1c‐based groups; for this review, the elevated‐normal group was classified as controlled low HbA1c (<6.5%), and normal‐elevated/elevated‐elevated as uncontrolled high HbA1c (≥6.5%). Calderon et al. [24], (Peru) assessed persistent dysglycemia during TB treatment across multiple visits; consistent dysglycemia indicated uncontrolled high HbA1c, while transient dysglycemia was considered controlled low HbA1c. Mahato et al. [28], (Nepal) compared TB outcomes in patients with and without DM; only data from TB‐DM patients were extracted, with outcomes stratified by HbA1c control. Udaykumar et al. [34], (India) used a cohort with nested case–control design to evaluate glycemic control and TB outcomes in programmatic settings; both supervised and unsupervised site data were included. Ghanta et al. [26], (India) reported mean HbA1c by TB outcome category but did not provide outcome counts by glucose control, thus their data were excluded from forest plots for treatment failure and cure.

### Quality assessment of included studies

NOS was used to assess the quality of each included study. There were six (46.2%) studies found to have good quality in terms of selection, comparability and outcome domains, while three (23.1%) had fair quality and the remaining four (30.8%) had poor quality. Comprehensive details of the quality assessment process, including individual study ratings and scoring criteria, were presented in Table 2.

**TABLE 2:** Quality assessment and scoring of included studies using the Newcastle‐Ottawa Scale (*n* = 12).

Study author, year	Selection domain				Comparability domain	Outcome domain			Overall quality
	1	2	3	4	5	6	7	8	
Calderon et al. [24], 2022		*	*	*	*	*	*	*	Good
Chiang et al. [25], 2015		*		*	*			*	Poor
Ghanta et al. [26], 2023	*	*	*	*	*	*	*	*	Good
Magee et al. [27], 2013			*			*	*	*	Poor
Mahato et al. [28], 2022		*	*	*	*	*	*	*	Good
Mahishale et al. [29], 2017	*		*	*	*		*	*	Good
Mi et al. [30], 2013	*	*	*	*	*			*	Fair
Nandhakumar et al. [31], 2013	*	*		*		*		*	Poor
Park et al. [32], 2012	*	*			*	*		*	Fair
Tabarsi et al. [33], 2014	*	*	*	*		*	*	*	Poor
Udaykumar et al. [34], 2022		*	*	*	*		*	*	Good
Yoon et al. [35], 2017			*	*	*		*	*	Fair

*Note*: 1: Representativeness of the exposed cohort, 2: Selection of the non‐exposed cohort, 3: Ascertainment of exposure. 4: Demonstration that outcome was not present at start of the study, 5: Comparability of cohorts on the basis of the design or analysis controlled for confounders, 6: Assessment of outcome, 7: Was follow‐up long enough for outcomes to occur, 8: Adequacy of follow‐up of cohorts. *Good quality: 3 or 4 stars in selection domain AND 1 or 2 stars in comparability domain AND 2 or 3 stars in outcome/exposure domain. *Fair quality: 2 stars in selection domain AND 1 or 2 stars in comparability domain AND 2 or 3 stars in outcome/exposure domain. *Poor quality: 0 or 1 star in selection domain OR 0 stars in comparability domain OR 0 or 1 stars in outcome/exposure domain.

### TB treatment outcomes

#### Treatment failure

**FIGURE 2:**
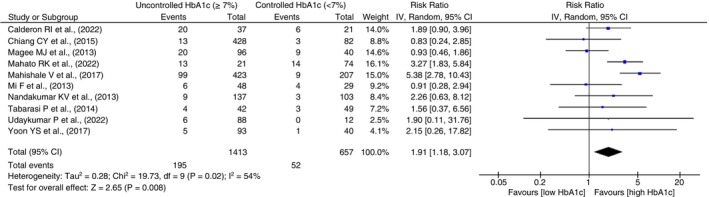
Forest plot comparing risk of treatment failure among patients with controlled and uncontrolled diabetes. CI, confidence intervals.

Ten studies examined treatment failure among TB patients with DM. Of 1413 patients with uncontrolled high blood glucose, 195 (13.8%) experienced treatment failure compared to 52 (7.9%) among 657 patients with controlled glucose. The random‐effects model indicated a significantly higher risk of treatment failure in patients with uncontrolled DM (RR = 1.91, 95% CI 1.81–3.07, *p* = 0.0008, *I* ^2^ = 54%, *p* = 0.02). Forest plots in Figure 2 display individual and pooled RRs with heterogeneity.

#### Treatment cure

**FIGURE 3:**
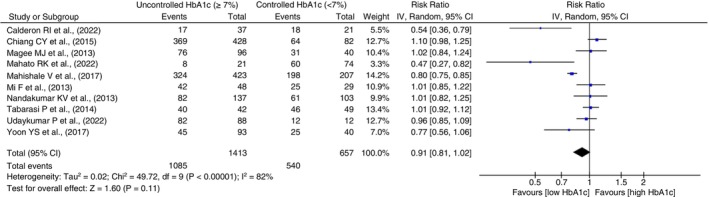
Forest plot comparing risk of treatment cure among patients with controlled and uncontrolled diabetes. CI, confidence intervals.

Ten studies assessed treatment cure rates. Among 1413 patients with uncontrolled glucose, 1085 (76.7%) were cured, compared to 540 (82.2%) out of 657 with controlled glucose. Although the likelihood of cure was found to be lower in participants with uncontrolled high HbA1c (RR = 0.91, 95% CI 0.81–1.02, *p* = 0.11, *I* ^2^ = 82%, *p* < 0.0001), the findings were not statistically significant. Forest plots in Figure 3 depict study‐level and pooled RRs.

#### Sputum smear positivity

**FIGURE 4:**
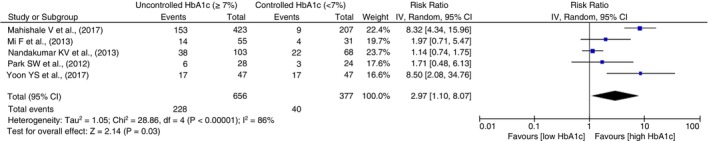
Forest plot comparing risk of sputum smear positivity at 3 months among patients with controlled and uncontrolled diabetes mellitus. CI, confidence intervals.

Five of 12 studies reported sputum smear conversion outcomes. Among 656 patients with uncontrolled DM, 228 (34.7%) remained smear‐positive at 3 months, compared to 40 (10.6%) of 377 patients with controlled DM. Uncontrolled high HbA1c was associated with a nearly threefold higher likelihood of smear positivity (RR = 2.97, 95% CI 1.10–8.07, *p* = 0.03, *I* ^2^ = 82%, *p* < 0.0001). Forest plots in Figure 4 illustrate individual and pooled associations.

#### Mortality

Four studies reported mortality during TB treatment. Among 700 patients with uncontrolled glucose, 53 (7.5%) died, compared to 21 (7.6%) of 274 with controlled glucose. No significant association between mortality and glucose control status was observed (RR = 0.96, 95% CI 0.37–2.51, *p* = 0.93, *I* ^2^ = 51%, *p* = 0.11). Forest plot in Figure S1 present mortality associations among two groups.

### Comparison of mean HbA1c and FBG

There was one study each, in which HbA1c [26] and FBG [27] glycemic markers in TB patients were compared between those with treatment success to those with treatment failure at 6 months. Patients who experienced treatment failure had significantly higher mean HbA1c levels, with a mean difference of 1.52% (95% CI 0.54–2.49, *p* = 0.002) shown as Figure S2. Similarly, mean FBG levels were also markedly elevated in the treatment failure group, with a mean difference of 54.20 mg/dL (95% CI 5.62–102.78, *p* = 0.03) given as Figure S3.

### Subgroup analysis

Subgroup analysis by age and sex was not performed as age and sex distribution was similar in almost all included studies, where the majority of the patients belonged to the 40–50 years age group, and almost all of the studies had a 60%–70% male population. The subgroup analysis was performed on the basis of methods used to capture the exposure and whether the studies reported adjusted analysis results. There were 6/10 studies assessing glucose levels by performing blood tests, while 4/10 considered readings from medical records. Similarly, there were 3/10 studies in which adjusted analysis results were reported, while 7/10 studies reported crude estimates. Overall, better pooled effects were observed after subgroup analysis of studies in which glucose testing was performed as compared to studies relying on medical records, as shown in Table S3.

#### Treatment failure

**FIGURE 5:**
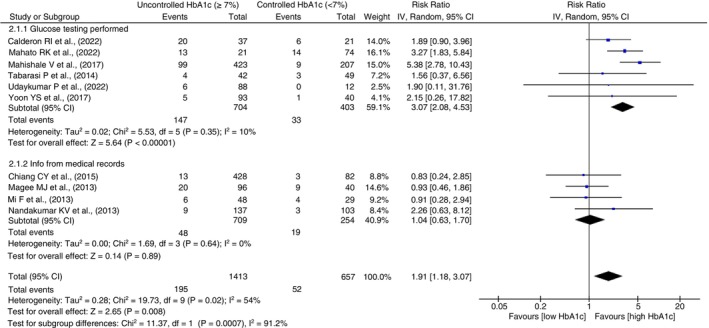
Subgroup analysis for treatment failure outcome using medical records and prospective testing of glucose levels to assess the exposure. CI, confidence intervals.

The subgroup analysis of six studies, in which glucose testing was performed to measure glucose control status, had a significantly better pooled association showing a three times higher risk of TB treatment failure due to uncontrolled high HbA1c (RR = 3.07, 95% CI 2.08–4.53, *p* < 0.0001, *I* ^2^ = 10%, *p* = 0.35). Studies using medical records for exposure information were found to have no significant pooled associations. A significant difference was observed between the pooled associations of these subgroups (*p* = 0.0007) for treatment failure, as shown in Figure 5. Subgroup analysis of treatment failure between adjusted and unadjusted estimates is given as Figure S4.

#### Treatment cure

**FIGURE 6:**
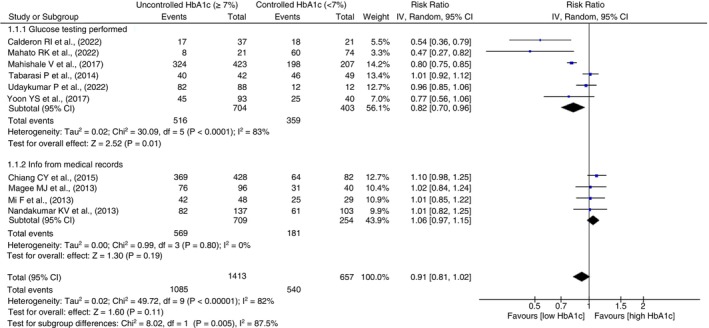
Subgroup analysis for treatment cure outcome using medical records and prospective testing of glucose levels to assess the exposure. CI, confidence intervals.

Subgroup of 6 studies, reported to perform glucose tests were found to have better significant pooled association showing 20% higher likelihood of treatment cure due to controlled HbA1c (RR = 0.82, 95% CI 0.70–0.96, *p* = 0.01, *I* ^2^ = 83%, *p* < 0.0001). In contrast, studies considering medical records reported no such association and the difference between these subgroup associations was statistically significant (*p* = 0.005) as shown in Figure 6. Subgroup analysis of treatment cure between adjusted and unadjusted estimates is given as Figure S5.

#### Sputum conversion

**FIGURE 7:**
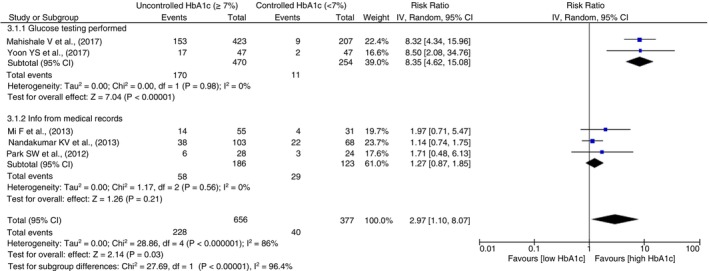
Subgroup analysis for sputum positivity outcome using medical records and prospective testing of glucose levels to assess the exposure. CI, confidence intervals.

A significant difference was observed between pooled subgroup associations (*p* = 0.0001) as shown in Figure 7. The pooled effect of three studies in which glucose testing was performed showed eight times higher risk of sputum positivity at 3 months of TB treatment in patients with uncontrolled high HbA1c as compared to controlled low HbA1c (RR = 8.35, 95% CI 4.62–15.08, *p* < 0.0001, *I* ^2^ = 0%, *p* = 0.98), while 3 other studies relying on medical records showed no such association. Subgroup analysis of sputum conversion between adjusted and unadjusted estimates is given as Figure S6.

### Sensitivity analysis

Sensitivity analysis was conducted for treatment failure, cure and sputum conversion outcomes by excluding the low‐quality studies and excluding studies causing excessive heterogeneity. Both sensitivity analyses resulted in stronger pooled effects compared to the overall meta‐analysis as shown in Table S4.

#### Treatment failure

The pooled effect increased to RR = 2.64 (95% CI 1.56–4.46, *p* = 0.0003, *I* ^2^ = 43%, *p* = 0.12) after excluding four studies with high risk of bias (Figure S7). Similarly, the pooled effect increased to RR = 2.05 (95% CI 1.43–2.94, *p* = 0.0001, *I* ^2^ 0%, *p* = 0.47) after excluding two studies causing heterogeneity (Figure S8).

#### Treatment cure

The pooled likelihood of treatment cure decreased from RR = 0.91 (95% CI 0.81–1.02, *I* ^2^ = 86%, *p* < 0.001) to RR = 0.81 (95% CI 0.70–0.95, *p* = 0.009, *I* ^2^ = 76%, *p* = 0.0009) after excluding high risk of bias studies (Figure S9). Sensitivity analysis performed for homogenous studies showed no effect of exposure on treatment cure outcome (Figure S10).

#### Sputum conversion

The pooled risk ratio increased to RR = 4.08 (95% CI 1.67–9.97, *I* ^2^ = 66%, *p* = 0.002) from the initial RR = 2.97 (95% CI 1.10–8.07, *p* = 0.002, *I* ^2^ = 86%, *p* = 0.03) by excluding biassed studies with a decrease in heterogeneity (Figure S11). Whereas, no significant effect was found between uncontrolled high blood glucose and sputum positivity analysing homogenous studies (Figure S12).

### Publication bias

Publication bias was assessed for all outcomes separately by constructing the funnel plots and applying Egger's test. For treatment failure outcome, the funnel plot (Figure S13) showed symmetric distribution of studies within the inverted funnel shape which showed no publication bias, this was also confirmed by Egger's regression test for funnel plot asymmetry (*b* = 1.0071, 95% CI −0.07 to 2.08, *p* = 0.46). On the contrary, for treatment cure and sputum conversion outcome, the funnel plots (Figures S14 and S15, respectively) showed asymmetric distribution of studies within inverted funnel shape, along with significant Egger's test *p*‐value further confirming the publication bias for studies reporting cure outcome (*b* = 0.13 95% CI −0.07 to 0.33, *p* = 0.012), while it was not performed for sputum conversion due to the number of studies being less than 10.

## DISCUSSION

This review included 12 articles examining the association between blood glucose control during TB treatment period in TB patients diagnosed with DM. Overall, the results suggest that uncontrolled high blood glucose levels (HbA1c >7%) nearly double the risk of TB treatment failure (RR = 1.91) and triple the risk of a positive sputum smear at 3 months (RR = 2.97), while having no significant effect on treatment cure compared to controlled blood glucose levels (HbA1c ≤7%) during TB treatment. Given the substantial heterogeneity observed, the overall certainty of the meta‐analysis findings was rated as low, warranting cautious interpretation and generalisation of the results. Although subgroup and sensitivity analyses were employed to explore and mitigate sources of heterogeneity and enhance the robustness and interpretability of the findings, these approaches could not fully account for the observed variability.

The findings of this review are consistent with reviews conducted and reported in the literature previously. There are two reviews [16, 17] published in literature exploring the associations between glucose control and TB treatment outcome in TB patients diagnosed with DM, the characteristics of whom are summarised in Table 3. The current review provided an update on association of glucose control on TB treatment outcomes among TB patients with DM. Shewade et al. [16], reported the review in 2017 with an aim to assess the effect of stringent glycemic control in comparison with poor glycemic control on unsuccessful TB treatment outcomes and also to compare effect of oral hypoglycaemic agents and insulin on the same. There were nine articles included in the review, results were reported qualitatively and meta‐analysis was not performed. Author stated that eight studies reported the effect of glycemic control on unsuccessful TB treatment outcomes where only two studies were labelled to have low risk of bias. The primary limitation was the inability to perform a meta‐analysis, likely due to the high degree of heterogeneity among the included studies. The authors advocated for high‐quality RCTs to better understand the impact of glycemic control therapies on DM management and ultimately leading to better TB treatment outcomes. Furthermore, the findings support currented recommendations for stringent glycemic control in TB‐DM patients and highlighted the need for programmatic guidelines to incorporate regular glycemic assessments [16].

**TABLE 3:** Summary of previous reviews.

Author, year	Objective	Methodological details	Pooled estimates	Limitations
Shewade et al. [16], 2019	To determine the effect of (i) glycemic control compared to poor glycemic control and (ii) insulin compared to oral drugs, on unsuccessful TB treatment outcome	*Eligibility criteria*: Interventional and/or cohort studies, between 1996 and 2017, participants of all ages, both genders, being treated for TB (pulmonary or extrapulmonary, new or retreatment) and diagnosed with diabetes with drug sensitive pulmonary TB *Exposure classes*: Stringent glycemic control (HbA1c <7%), less stringent glycemic control (HbA1c <8%), poor glycemic control (HbA1c >8%) *Time for exposure measurement*: At baseline or any time during TB treatment *Primary outcome*: TB treatment outcomes (favourable, unfavourable) at end of treatment *Included studies*: 9 cohort studies *RoB*: New Castle Ottawa quality assessment scale was used to assess quality, two studies were reported to have low risk of bias *Meta‐analysis*: Not performed	Not applicable	The review included studies up to 2017, therefore an updated review is needed. The authors could not perform meta‐analysis
Zhao L et al. [17], 2024	To assess the role of glycemic control in improving the TB treatment outcomes among TB patients with DM	*Eligibility criteria*: RCTs or analysis of RCTs or cohort studies assessing effect of glucose control on TB treatment outcomes in TB patients with DM, studies reporting radiological findings, treatment success, sputum conversion and death *Exposure classes*: Optimal glucose control, Poor glucose control *Time for exposure measurement*: Not mentioned *Primary outcome*: Radiological findings, treatment success, sputum positivity and mortality. *Included studies*: 9 cohort studies *RoB*: New Castle Ottawa quality assessment scale was used to assess quality, two studies were reported to have low risk of bias *Meta‐analysis*: Pooled risk ratios (RRs) with 95% CI using inverse variance–weighted random‐effects model. Moderate heterogeneity reported.	Better TB treatment outcomes (RR 1.13, 95% CI 1.02–1.25; *p* = 0.02; *I* ^2^ = 65%), reduced sputum positivity (RR 0.23, 95% CI 0.09–0.61; *p* = 0.003; *I* ^2^ = 66%) and fewer cavitary lesions (RR 0.59, 95% CI 0.51–0.68; *p* < 0.001; *I* ^2^ = 0%) in patients with optimal glucose control. No significant differences in terms of mortality and lobar involvement.	The review was published in 2024 but latest studies from 2017 onwards were not included. Patient characteristics not reported from individual studies. No subgroup analysis or sensitivity analysis was performed and reported. Only generic interpretation and reasons of heterogeneity reported. Publication bias not properly assessed and reported.

Abbreviations: CI, confidence interval; DM, diabetes mellitus; RCTs, randomised controlled trials; TB, tuberculosis.

Zhao et al. [17], recently reported a review in 2024, with an aim to assess the role of glycemic control in improving the TB treatment outcomes among TB patients with DM. Key findings from seven observational studies involving 6919 participants highlight that optimal glucose control significantly enhances TB treatment outcomes (RR 1.13, 95% CI 1.02–1.25), reduces sputum positivity (RR 0.23, 95% CI 0.09–0.61), and lowers the risk of cavitary lesions (RR 0.59, 95% CI 0.51–0.68). However, no significant differences were observed in mortality or specific radiologic outcomes such as multi‐lobar or isolated lobe involvement. The primary limitations of the review include the exclusion of studies published between 2020 and 2023, limited exploration and explanation of heterogeneity, lack of interpretation of results in the context of this heterogeneity, and the absence of subgroup and sensitivity analyses. In conclusion, the authors emphasised the need for early detection of DM in TB patients and highlighted the potential of optimal glucose control in reducing disease severity, transmission and overall burden [17].

Most of the studies included in current review included both TB patients with and without DM, although keeping in view the objectives of the current review only relevant data was extracted and analysed. Some of the studies reported to extract exposure information from while in other research studies the exposure was assessed longitudinally at baseline, during or at end of TB treatment. Most of the studies reported using HbA1c measurements, that is more accurate, to assess the extent of exposure, while a few [30, 31] relied on fasting or random blood glucose tests. One study included both pulmonary and extrapulmonary TB patients, while other included only pulmonary TB patients. None of the studies reported enough data on TB and DM‐related covariates and confounders therefore, meta‐regression could not be performed.

This review provides an updated synthesis of the existing literature, with several notable strengths. These include the incorporation of five recently published studies, a systematic comparison with prior reviews, and subgroup analyses based on methods used to assess glucose exposure and reported adjusted analysis. We also conducted two types of sensitivity analyses, one based on risk of bias and another on heterogeneity. Publication bias was evaluated both visually using funnel plots and statistically via Egger's test. In addition, we used the recommended NOS to assess study quality across the domains of selection, comparability and outcome. Limitations include minor deviations from the PROSPERO‐registered protocol, specifically in the inclusion criteria and risk of bias assessment tool made on the recommendation of the PhD advisory panel during the conduct of this PhD‐led review. Additional limitations include the inability to report adjusted pooled effect estimates due to insufficient data in the primary studies. Furthermore, none of the included studies provided outcome data stratified by age or gender, which restricted our ability to conduct meaningful subgroup analyses based on these variables. Substantial heterogeneity was observed, likely due to differences in study design, populations and methods of exposure and outcome measurement. These variations reduce the certainty and limit the generalisability of the findings. Although sensitivity analyses were conducted to explore and partially address this heterogeneity, methodological inconsistencies across studies remain an inherent limitation. Therefore, results should be interpreted with caution, particularly when applied to diverse contexts and populations.

It is recommended that future research aim to report and analyse HbA1c levels as a continuous instead of a binary variable, adjusted results for potential confounders, and provide demographic and contextual details of participants as possible sources of heterogeneity.

## CONCLUSION

A low certainty evidence is found on the association of uncontrolled glycemic status with TB treatment failure and 3‐month sputum smear positivity. This review highlights the need for more research on glycaemic control outcomes in TB patients to better understand its true impact on treatment outcomes.

## CONFLICT OF INTEREST STATEMENT

There is no conflict of interest to be reported for any mentioned authors.

## Supporting information


**Data S1.** Supporting Information.

## Data Availability

The data can be accessed by requesting the first author.
